# Supplementary material for: Hirudo verbana Microbiota Dynamics: A Key Factor in Hirudotherapy-Related Infections?
Source: Microorganisms. 2025 Apr 16;13(4):918. doi: 10.3390/microorganisms13040918 (PMC12029263; doi:10.3390/microorganisms13040918)
Supplement: Supplementary file 1 [file microorganisms-13-00918-s001.zip › microorganisms-3572399-supplementary.pdf]

Supplementary Table S1. 16S rRNA V3-V4 region sequences obtained from *H. verbana* in Türkiye.

Sample-ID	Input	Filtered	Input passed filter (%)	Denoised	Non-chimeric	Input non-chimeric (%)
J_1	113,313	103,082	90.97	101,387	94,931	83.78
J_2	129,146	115,831	89.69	114,805	95,474	73.93
J_3	133,004	120,427	90.54	119,183	101,594	76.38
J_4	142,755	129,119	90.45	127,817	102,720	71.96
J_5	133,754	119,780	89.55	114,831	106,324	79.49
J_6	120,194	105,307	87.61	104,212	86,171	71.69
J_7	88,787	78,217	88.10	76,915	65,937	74.26
J_8	109,860	99,813	90.85	99,014	85,051	77.42
J_9	114,707	106,041	92.45	105,204	97,967	85.41
J_10	109,130	100,132	91.75	97,491	82,111	75.24
J_11	127,370	117,877	92.55	115,812	97,322	76.41
J_12	142,056	131,362	92.47	129,793	104,058	73.25
Mean	122,006	110,582	90.57	108,872	93,305	76.60

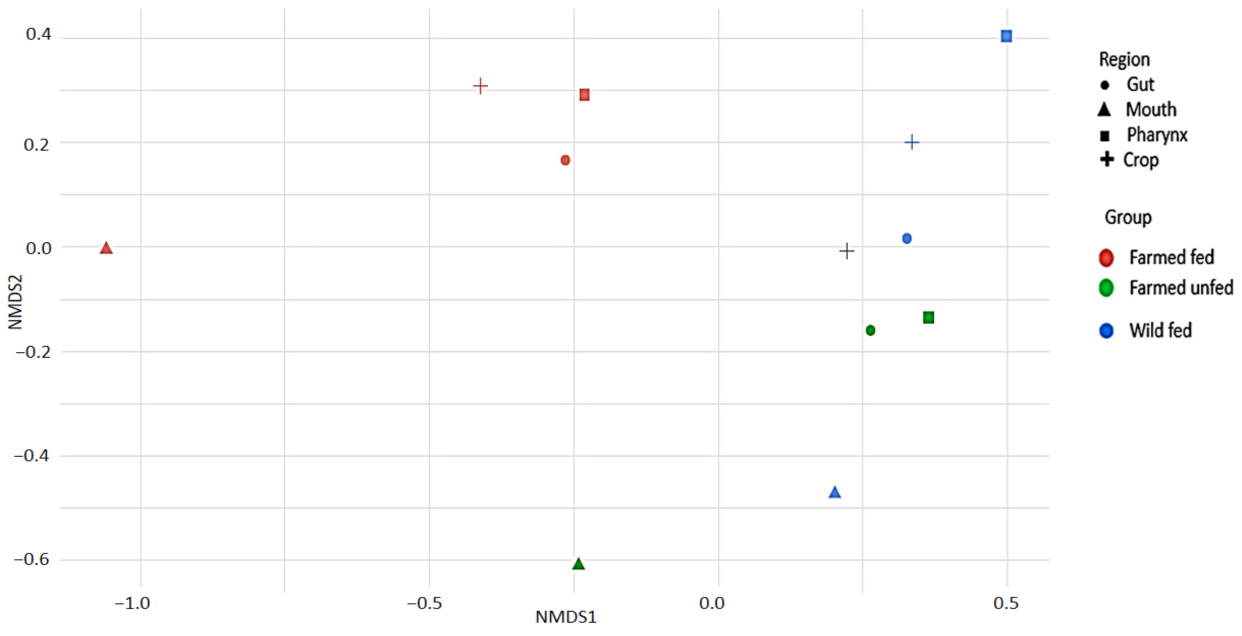

Supplementary Figure S1. The NMDS plot illustrates a scattered distribution of the microbial communities across the regions of the digestive tract of the three leech groups, suggesting a lack of clear clustering or separation among the groups.
